# Blacks and whites in the Cuba have equal prevalence of hypertension: confirmation from a new population survey

**DOI:** 10.1186/1471-2458-13-169

**Published:** 2013-02-24

**Authors:** Pedro Ordúñez, Jay S Kaufman, Mikhail Benet, Alain Morejon, Luis C Silva, David A Shoham, Richard S Cooper

**Affiliations:** 1 Project for Chronic Disease Prevention and Control, Pan American Health Organization, 20037, Washington DC, USA; 2 Centro de Estudios sobre Enfermedades Crónicas, Universidad de Ciencias Médicas, 55100, Cienfuegos, Cuba; 3 Department of Epidemiology, Biostatistics, and Occupational Health, McGill University, 1020 Pine Ave West, H3A 1A2, Montreal, QC, Canada; 4 Centro Nacional de Información de Ciencias Médicas, La Habana, Cuba; 5 Department of Preventive Medicine and Epidemiology, Loyola University Stritch School of Medicine, 60153, Maywood, IL, USA

**Keywords:** Blood pressure, Cuba, Ethnic groups, Hypertension, Stress

## Abstract

**Background:**

The excess burden of hypertension among blacks has been a prominent feature of the heath disparities literature, and many scientists presume it to be a stable and inevitable phenomenon. The underlying causes of this disparity can only be disentangled in a setting in which the population does not experience racial stratification of socioeconomic opportunities. While such conditions of racial equality remain uncommon, they may be approximated in Cuba, a country with a persistent policy of social inclusion over the last 5 decades.

**Methods:**

We report on a 2010–2011 stratified probability sample of those aged 15–74 years from the urban population of Cienfuegos in central Cuba. A total of 1496 adults (880 women and 616 men) were recruited and assessed for blood pressure and anthropometrics according to standardized protocols, as well as medication use, educational attainment and observed skin tone (dichotomized into “black” and “white”). Weighted tabular and regression analyses were conducted to estimate adjusted prevalences of hypertension (> 140/90 mmHg) and adjusted prevalence odds ratios for contrasts between the two skin color groups.

**Results:**

Mean pressures were higher for men than for women, but overall did not differ importantly between racial groups. About half of all diagnosed hypertensive men were on medication, a proportion that did not vary by racial group. For women, however, adjusted prevalence was somewhat higher among blacks, and treatment and control rates were also somewhat advantaged for white women.

**Conclusions:**

Overall, skin color was unrelated to mean blood pressure or hypertensive status in this population, although among women specifically some racial advantage appears evident in adjusted prevalence and control, and should be investigated further. The overall null result suggests that Cuba may exemplify the social conditions in which racial excess in hypertension, characteristic of much of the western world, is not a necessary reality.

## Background

Since the early decades of the 20^th^ century one of the most prominent features of health disparities in the US has been the excess burden of hypertension endured by blacks compared to whites [1]. Despite considerable effort, it has not yet been possible to define the environmental exposures which adequately account for the differential risk, nor have recent efforts in molecular epidemiology provided sufficient evidence to support a genetic explanation [2,3]. Environmental factors underlying hypertension are difficult to characterize, and consistently explain a small portion on an individual’s risk [4,5]. Likewise, genetic studies are still in their infancy, with only 1-2% of the variance associated with known polymorphisms [6].

One line of argument regarding racial/ethnic differentials in hypertension holds that the cumulative effects of social disadvantage (including nutritional and other behavioral factors, as well as psychosocial stressors resulting from discrimination) would explain the observed excess risk if it could be measured [7]. Epidemiological survey methods are inadequate to capture most of these exposures, however, especially in the psychological domain [8]. In support of this argument, a social class gradient is often observed in hypertension, and clear geographic differences in hypertension burden have been well documented [9].

The “social-structural” theory, which invokes the net effect of discrimination, can only be tested under counterfactual conditions of social equality between blacks and whites. Unfortunately these conditions cannot be found in North America, the UK, or Brazil, where the vast majority of persons in the African Diaspora live. We previously examined racial hypertension differentials in Cuba, a country with a persistently implemented policy of social inclusion stemming from the 1959 revolution, and we found consistent evidence of a diminished prevalence gap between blacks and whites compared to the US [10,11]. We report here findings from a new survey in Cuba confirming an absence of substantial racial differentials in blood pressure.

## Methods

The city of Cienfuegos is located in the south and center of the main island of Cuba; with an adult population of 161,828 inhabitants it is the 10^th^ largest in the country [12]. Cienfuegos is the only city in Cuba with population-based data on major cardiovascular risk factors obtained systematically over the last three decades [13]. The Cienfuegos survey was conducted during 2010–2011, based on a probability sample from the urban population aged 15–74 years, and included 1496 adults. The population of Cienfuegos is similar to the general Cuban population in terms of age and race distribution and cardio-vascular mortality (Additional files 1 and 2).

With assistance from the Provincial Department of Statistics and Census, a multi-stage, probability sample was selected from urban Cienfuegos to provide 180–200 persons in gender by 10-year age groups. The sampling design aimed to recruit roughly 2200 adults selected with equal probability within each of the defined age-gender groups. For population estimations, the proper weights were computed taking into account the Cienfuegos city resident adult population as most recently estimated in July 2008 [14]. The 2011 survey protocol incorporated portions of a questionnaire developed in a Pan American Health Organization project for risk factor surveillance and intervention for non-communicable diseases [15].

Data were collected by self-report on a variety of health-related conditions, and anthropometrics were measured directly. Body mass index was calculated as weight in kilograms divided by height in meters squared. Blood pressure was measured three times at the same sitting using a mercury manometer by trained personnel, and analyses were based on the mean of the last two readings. No terminal digit was assigned more than 25 percent of the values, suggesting an adequate measurement technique. Blood pressure medication use was verified by the physician, who examined the pill bottles. Assignment to the categories of white (n = 1077), black (n = 131), and “mulatto” (i.e. mixed race) (n = 288) was made by trained professional interviewers who had participated in the most recent national census. The black and mixed-race categories were combined in the primary analyses, reflecting the standard North American convention of racial dichotomy. Education was dichotomized at “pre-universitario” and above, roughly equivalent to more than high school in the US.

Hypertension was defined as a systolic blood pressure (SBP) of ≥140 mmHg, a diastolic blood pressure (DBP) of ≥90 mmHg, or current treatment with antihypertensive agents. “Control in the population” was defined as the percentage of all hypertensives with a SBP of <140 mmHg and a DBP of <90 mmHg, irrespective of whether they had been previously diagnosed or whether treatment had been recommended. “Control in treated patients” was defined as the number of treated patients achieving the same goal (<140/90 mmHg) divided by the number of patients on treatment.

Descriptive analysis was conducted of means and frequencies by gender and skin-color group taking into account the complex sampling design and using EPIDAT, a freely distributed software package developed by the Service of Epidemiology of the Dirección Xeral de Saúde Pública from the “Consellería de Sanidade” (Xunta de Galicia, Spain) and the Health Situation Analysis Program (SHA) of the Pan American Health Organization (http://www.paho.org/english/sha/epidat.htm). Unconditional logistic regression analyses were used to estimate the ratio of prevalence odds (POR) adjusted for multiple covariates. Weighted prevalences were obtained for estimations of the awareness, treatment and control rates at population level.

The study protocol received ethics review and approval from the Comité de Ética de la Investigación Científica (CEIC) of the University of the Medical Sciences of Cienfuegos (UCMC). Participants signed informed consent statements when they enrolled, and again prior to each clinic visit.

## Results

A total of 2193 individuals completed the questionnaire, 1496 of which also participated in the clinical examination (68%). There was no evidence of differential response by measured characteristics of the participants. A total of 880 women were measured, along with 616 men. The combined non-white group (referred to henceforth as “blacks”) comprised 27.9% of the weighted sample, which matches the most recently available census data. Mean pressures were higher for men than for women (Table 1), as were waist circumferences and prevalence of smoking. Women had higher prevalence of self-reported diabetes.

**Table 1:** Descriptive characteristics of the population sample (means, percentages, and 95% confidence intervals), Cienfuegos, 2011

	**Whites**		**Blacks**	
	**Men (N =456)**	**Women (N =621)**	**Men (N = 160)**	**Women (N = 259)**
Age (years)	40.9 (39.5, 42.3)	41.3 (40.1, 42.5)	38.0 (35.6, 40.4)	39.3 (37.4, 41.2)
Systolic blood pressure (mmHg)	123.3 (121.8, 124.8)	116.7 (115.4, 118.0)	122.2 (119.7, 124.7)	117.8 (115.5, 120.1)
Diastolic blood pressure (mmHg)	81.9 (80.7,83.0)	76.3 (75.4, 77.2)	81.0 (78.9, 83.0)	77.4 (75.8, 79.0)
Percent hypertensive ^a^	35.3 (31.0, 39.8)	26.6 (23.6, 29.9)	30.9 (24.1, 38.7)	31.0 (25.8, 36.7)
Body mass index (kg/m^2^)	25.7 (25.2, 26.2)	26.2 (25.7, 26.7)	25.7 (24.1, 27.3)	25.9 (25.0, 26.9)
Percent obesity (BMI ≥ 30 kg/m^2^)	15.7 (12.4, 19.7)	20.1 (16.9, 23.6)	14.4 (9.5, 21.3)	18.0 (13.7, 23.4)
Waist circumference (cm)	89.3 (88.1, 90.4)	83.7 (82.7, 84.7)	86.4 (84.3, 88.5)	82.1 (80.6, 83.7)
Percent current smokers	25.9 (21.8, 30.4)	19.4 (16.3, 23.0)	30.1 (23.0, 38.3)	22.5 (17.6, 28.4)
Percent with self-reported diabetes	4.6 (3.0, 6.9)	8.2 (6.3, 10.6)	3.6 (1.7, 7.5)	6.3 (4.1, 9.8)
Percent with post Secondary Education	71.1 (66.5, 75.3)	67.4 (63.6, 71.0)	70.1 (61.9, 77.2)	68.1 (61.9, 73.7)

^a^ SBP/DBP ≥ 140/90 mmHg or currently taking antihypertensive medication.

Mean systolic and diastolic blood pressures did not differ importantly between racial groups with both genders combined (Table 2), nor did prevalence of hypertension differ substantially (Table 3). Further adjustments for other measured covariates produced no discernible racial excess of hypertension. Stratified by gender, this remained true for men, although in the female stratum blacks had over 4 percentage point higher prevalence of hypertension, which was a significant excess in adjusted models (Table 3).

**Table 2:** Blood pressure (mmHg) by sex and skin color group, Cienfuegos, 2011

**Sex**	**N**	**Whites**	**N**	**Blacks**	**p-value**
	**Systolic blood pressure (weighted mean and 95% CI)**				
Men	456	123.3 (121.8, 124.8)	160	122.2 (119.7, 124.7)	0.46
Women	621	116.7 (115.4, 117.9)	259	117.8 (115.4, 120.1)	0.42
Total	1077	120.1 (119.1, 121.1)	419	119.8 (118.1, 121.6)	0.89
	**Diastolic blood pressure (weighted mean and 95% CI)**				
Men	456	81.9 (80.7, 82.9)	160	81.0 (78.9, 82.9)	0.41
Women	621	76.3 (75.4, 77.1)	259	77.4 (75.8, 78.9)	0.23
Total	1077	79.1 (78.4, 79.9)	419	79.1 (77.8, 80.3)	1.00

**Table 3:** Age-adjusted prevalence and 95% confidence intervals of hypertension in blacks and whites, Cienfuegos 2011

**Group**	**N**	**Prevalence (95% CI)**^**1**^	**POR**^**2**^**(95% CI)**	**POR**^**3**^**(95% CI)**	**POR**^**4**^**(95% CI)**
Total	1496	31.0 (28.8, 33.3)			
Men	616	34.1 (30.5, 37.9)	1.3 (1.1,1.6)	1.5 (1.1, 1.9)	2.1 (1.6, 3.0)
Women	880	27.9 (25.0, 30.6)	1.0	1.0	1.0
Blacks	419	31.0 (26.7, 35.7)	1.0 (0.8,1.3)	1.2 (0.9, 1.6)	1.2 (0.9, 1.6)
Whites	1077	31.0 (28.4, 33.8)	1.0	1.0	1.0
Men					
Blacks	160	30.9 (24.1, 38.7)	0.8 (0.5,1.2)	0.9 (0.6, 1.5)	0.9 (0.6, 1.5)
Whites	454	35.3 (31.0, 39.8)	1.0	1.0	1.0
Women					
Blacks	259	31.0 (25.8, 36.7)	1.2 (0.9,1.7)	1.5 (1.1, 2.3)	1.7 (1.1, 2.5)
Whites	621	26.6 (23.6, 29.9)	1.0	1.0	1.0

^1^ SBP ≥ 140 mmHg or DBP ≥ 90 mmHg or currently taking antihypertensive medication.

^2^ Crude Prevalence Odds Ratio.

^3^ Age-Adjusted Prevalence Odds Ratio.

^4^ Prevalence Odds Ratio adjusted for age, education, BMI, waist circumference, alcohol intake, and exercise.

There was no detectable difference in awareness of hypertension by racial group (Table 4), although women showed higher levels of awareness than men overall. About half of all diagnosed hypertensive men were on medication, a proportion that did not vary by racial group. For women, however, the treatment rate was higher than for men, and higher for white women than for black women. Likewise, control in the population did not differ by racial group among men, but was superior in white women compared to black women. Whites showed better control among the treated in both gender strata (Table 4).

**Table 4:** Age-adjusted prevalences and 95% confidence intervals of awareness, treatment and control of hypertension in blacks and whites, by sex, Cienfuegos 2011

	**Men**				**Women**			
	**Whites (N =193)**	**Blacks (N =62)**	**Difference (95% CI)**	**p**	**Whites (N = 224)**	**Blacks (N =107)**	**Difference (95% CI)**	**p**
Aware of being hypertensive: % (95% CI)	54.7 (46.9,62.3)	55.3 (41.2, 68.6)	0.6 (0.4, 0.8)	0.99	78.0 (71.3, 83.5)	71.8 (61.0, 80.6)	6.2 (6.1,6.3)	0.16
Currently taking medication: % (95% CI)	42.9 (35.5, 50.6)	47.7 (34.3, 61.5)	4.8 (4.6, 5.0)	0.85	71.4 (64.3, 77.6)	60.6 (49.9, 70.4)	10.8 (10.7,10.9)	0.05
Control in the population^1^: % (95% CI)	21.4 (15.8, 28.4)	17.0 (9.2, 29.3)	4.4 (4.1, 4.7)	0.55	37.8 (31.2, 44.9)	24.6 (17.0, 34.1)	13.2 (13.0,13.4)	0.01
Control among the treated^2^: % (95% CI)	50.0 (38.6, 61.4)	35.7 (20.0, 55.1)	14.3 (14.1,14.5)	0.04	53.0 (44.8, 61.0)	40.5 (28.9, 53.3)	12.5 (12.3,12.7)	0.03

^1^ Percent of all hypertensives with measured blood pressure < 140/90 mmHg.

^2^ Percent of treated hypertensives with measured blood pressure < 140/90 mmHg.

## Discussion

The health disadvantage of US blacks in comparison to the majority population extends across the entire spectrum of common conditions and the combined effects of economic and racial discrimination are widely recognized as the over-riding cause [16]. Hypertension is more commonly seen as a special case, however, and “racial-genetic” explanations have therefore gained much wider acceptance [17]. Racial differences in the primary risk factors, such as obesity, nutrition, alcohol or physical activity, are modest at best and cannot account for the 5–7 mmHg excess systolic blood pressure observed in US adults [18]. While the experience of racial discrimination, through conscious or unconscious mechanisms, has been proposed as the crucial missing risk factor, empirical evidence to support this hypothesis has always been elusive [19]. The Cuban experience, now further replicated in the data presented in this report, offers support to the claim that unmeasured social-structural factors account for higher blood pressures in US blacks, and provide *prima facie* evidence against the necessity of any vague genetic hypothesis for increased susceptibility.

An unexplained feature of the data presented here is the pattern of gender effects, specifically the excess hypertension risk in black women after covariate adjustment. Although the mean blood pressure differences among women by ethnicity were modest (1 mmHg for both systolic and diastolic readings), the age-adjusted adjusted hypertension prevalence differential was more pronounced (31.0 percent vs. 26.6 percent), and increased with further adjustment. This is a consistent pattern observed in previous surveys as well [11], and along with notable racial differences in control rates by gender suggests that the intersection of race and gender in this society is still a topic that requires deeper understanding [20].

The implications of this finding for Cuba itself are relevant to its overall policy emphasis on primary prevention and public health, as well as health and social equity along numerous dimensions. The pattern observed here contrasts with other Western Hemisphere nations where evidence suggests a more pronounced racial disparity [21-23]. It is therefore tempting to interpret this difference in light of the consciously anti-racist interventions made by the Cuban state over the last 50 years [24]. The challenge for local policy makers, however, will be to maintain these achievements under whatever economic or sociopolitical changes occur, and to encourage greater population surveillance of this type to establish equity benchmarks that can be monitored and defended.

The broader implications for understanding hypertension etiology and the comparison with entrenched racial disparities in the US are less clear. As in all of Latin America, admixture between persons of African and European descent in Cuba has been common since the early days of colonization [25]. Admixture could therefore dilute any putative genetic effect; in the absence of known loci that significantly influence risk of hypertension this question cannot be pursued directly. A plausible role for admixture, however, requires that a relatively small number of loci have a large effect on the trait, and we now have persuasive evidence that genetic predisposition for blood pressure is spread across hundreds of weakly acting polymorphisms [6].

Furthermore, it is crucial to recognize that relative to other world populations, US whites are in fact the exception, not US blacks. Thus, compared to many European countries, particularly Eastern Europe, Russia and Finland, blood pressure levels in US blacks are the same or substantially lower [26]. A standardized international comparative study also demonstrated that blood pressures in the English-speaking Caribbean are similar to those found among US whites [27]. The salience of these international comparisons is reinforced by data on mortality rates from stroke [28]; US whites have among the lowest death rates from stroke in the world, while 10-fold higher rates are found in Russia [29]. The perception of unusual hypertension risk among US blacks is therefore in part the result of a myopic interpretation of a comparison restricted to US whites.

## Conclusion

The data presented here of course have important limitations. The sample size in this study is relatively small, and restricted to a single urban center. Racial group was assigned based on observations by interviewers and, particularly in the Spanish Caribbean, is a categorization scheme with ambiguous borders. Nonetheless, in the context of a growing appreciation for the unique social environment created in Cuba for the study of racial disparities [30], this study confirms that the commonly observed black excess in blood pressure is neither a fixed nor a natural phenomenon, and suggests that the study of this particular sociopolitical environment may offer a unique lens into the etiology of the racial disadvantage that plagues the developed world.

## Abbreviations

mmHg: Millimeters of mercury; US: United States; SBP: Systolic blood pressure; DBP: Diastolic blood pressure; POR: Prevalence odds ratio

## Competing interests

The authors declare no financial conflict of interests. Although the conduct of the survey was supported by the Ministry of Public Health of Cuba, authors received no additional financial payments for the analyses reported here, and the Ministry had no role in the analysis or interpretation of the data. JSK reports support from the Canadian Research Chairs Program, which also defrayed the publication charges for the article but which had no influence on the conduct of the study or on the content of the paper.

## Authors’ contributions

PO, JSK, DAS and RSC drafted the manuscript. PO, MB, AM and LCS designed the study and oversaw the collection of the data. JSK and LCS provided statistical analysis and support. All authors provided critical comments on the manuscript, participated in revisions of the text, and approved the final version of the manuscript.

## Pre-publication history

The pre-publication history for this paper can be accessed here:

http://www.biomedcentral.com/1471-2458/13/169/prepub

## Supplementary Material

Additional file 1 Age-adjusted heart disease mortality rate per 100,000 inhabitants in Cuba and its provinces, 2011. Click here for file

Additional file 2 Population pyramid from Cienfuegos and Cuba, 2002. Click here for file
