# Supplementary material for: Blacks and whites in the Cuba have equal prevalence of hypertension: confirmation from a new population survey
Source: BMC Public Health. 2013 Feb 24;13:169. doi: 10.1186/1471-2458-13-169 (PMC3635894; doi:10.1186/1471-2458-13-169)
Supplement: Additional file 1 — Age-adjusted heart disease mortality rate per 100,000 inhabitants in Cuba and its provinces, 2011. [file 1471-2458-13-169-S1.docx]

Appendix 1:

| **Cuba** | **103.5** |
| --- | --- |
| Pinar del Rio | 106.1 |
| Artemisa | 87.6 |
| La Habana | 121.9 |
| Mayabeque | 112.2 |
| Matanzas | 122.5 |
| Villa Clara | 90.6 |
| **Cienfuegos** | **102.6** |
| S. Spíritus | 87.3 |
| Ciego de Ávila | 94.2 |
| Camaguey | 96.4 |
| Las Tunas | 97.4 |
| Holguín | 89.8 |
| Santiago de Cuba | 98.4 |
| Guantanamo | 95.4 |
| La Isla | 108.4 |

Source: Cuba. Ministerio de Salud Pública. Anuario Estadístico 2011. Ministerio de Salud Pública. 2012, pp. 38-39 <http://files.sld.cu/dne/files/2012/04/anuario-2011-e.pdf>
