# Supplementary material for: Blacks and whites in the Cuba have equal prevalence of hypertension: confirmation from a new population survey
Source: BMC Public Health. 2013 Feb 24;13:169. doi: 10.1186/1471-2458-13-169 (PMC3635894; doi:10.1186/1471-2458-13-169)
Supplement: Additional file 2 — Population pyramid from Cienfuegos and Cuba, 2002. [file 1471-2458-13-169-S2.docx]

2011 Age-Adjusted Heart Disease Mortality Rates (per 100,000 per year) for Cuba Overall and for Regions, including Cienfuegos
